# Quality of Life in COVID-19 Outpatients: A Long-Term Follow-Up Study

**DOI:** 10.3390/jcm11216478

**Published:** 2022-10-31

**Authors:** Vincent Tarazona, David Kirouchena, Pascal Clerc, Florence Pinsard-Laventure, Bastien Bourrion

**Affiliations:** 1 Department of Family Medicine, Faculty of Health Sciences Simone Veil, University Versailles-Saint-Quentin-en-Yvelines (UVSQ), 78180 Montigny le Bretonneux, France; 2 Clinical Epidemiology and Ageing Unit, University Paris-Est Creteil (UPEC), 94000 Créteil, France; 3 Center for Research in Epidemiology and Population Health, French National Institute of Health and Medical Research (INSERM), University Paris-Saclay, UVSQ, Paul-Brousse Hospital, 94800 Villejuif, France

**Keywords:** COVID-19, long COVID outpatients, quality of life

## Abstract

Background: The long-term issues faced by COVID-19 survivors remain unclear. Symptoms may persist for several months, even in non-hospitalized patients, probably impacting the quality of life. Objective: To assess the health-related quality of life of outpatients one year after SARS-CoV-2 infection. Design, Settings, and Participants: This prospective multicentre study, conducted in France from February 2020 to February 2022, compared 150 COVID-19 cases (PCR+ and/or CT scan+) and 260 controls (PCR-) selected from a database of four COVID centres. Main outcomes: Health-related quality of life assessed using the EQ-5D-5L scale. Results: COVID-19 outpatients (n = 96) had significantly lower health-related quality of life than controls (n = 81) one year after SARS-CoV-2 infection: the EQ-5D-5L index averaged 0.87 in cases and 0.95 in controls (*p* = 0.002); the EQ- VAS averaged 78 in cases and 86.7 in controls (*p* < 0.001). This alteration in quality of life was more intense in the areas of pain or discomfort and daily activities. Conclusions: This study is the first to show an alteration in the quality of life of COVID-19 outpatients after one year. Appropriate guidance and community rehabilitation programs are required for outpatients with persistent symptoms of COVID-19. Research must continue to confirm these results in larger cohorts.

## 1. Introduction

The coronavirus disease (COVID-19) pandemic has changed humanity’s way of life. Since the spring of 2020, there have been more than 30 million confirmed cases in France, causing more than 150,000 deaths [1]. However, death is not the only outcome to measure. Many patients had a slow and painful return to their former state of health [2]. Of specific concern, this finding was not limited to patients recovering from severe COVID-19 (requiring admittance to intensive care unit [ICU]), but also to patients with mild or moderate COVID-19. The sharing of personal experiences of patients and medical professionals [3,4,5] led to the emergence of the term “long COVID”, used to describe the disease in people who report lasting effects of the infection [6].

The persistence of symptoms has been reported in survivors of SARS-CoV-1, with a deficiency in the pulmonary diffusion capacity of carbon monoxide (DLCO) accompanied by significantly lower quality of life and exercise capacities [7]. Recent publications tend to confirm the persistence of symptoms several months after the end of the acute phase of the infection in a considerable number of patients [8,9], and non-hospitalised patients in particular [10]. These patients’ feelings of hopelessness are compounded by uncertainty expressed by medical professionals [11]. They feel ignored [12], suggesting that the opinion of the public, the media, and health professionals on COVID is focused only on one of two extremes: mild or severe. These individuals ask their general practionner to validate their symptoms and show empathy [13]. Patients often attempted to “manage” their symptoms independently, without seeking medical advice.

A few studies focusing on hospitalised patients have shown a significant impairment in the quality of life assessed by the EQ-5D-5L scale, up to approximately three months of follow-up [14,15]. However, the impact of persistent COVID-19 symptoms on quality of life in non-hospitalised patients may be underestimated: the consequences of viral infections remain poorly understood. This assessment also helps healthcare professionals to identify risk factors and recognise areas that can be improved to relieve patient symptoms [16].

This study aimed to assess the health-related quality of life of outpatients one year after SARS-CoV-2 infection.

## 2. Methods

### 2.1. Design, Settings, and Population

This prospective, multicentre, community-based study, conducted in France from February 2020 to February 2022, used a database created by the Department of General Medicine of Simone Veil University. This database combines the demographic and clinical data of patients who consulted one of the four main COVID outpatient centres in the territory of the Yvelines (78) during the first wave of the epidemic: Les Mureaux, Mantes-La -Jolie, Trappes, and Triel-sur-Seine.

This database included 522 patients who underwent polymerase chain reaction test (PCR), of whom approximately 30% were positive. In addition, out of 116 chest computed tomography (CT) scans performed, approximately 75% were positive for COVID-19.

The inclusion criteria included all the adult patients who consulted in one of these four centres between February and September 2020. COVID patients were included based on positive PCR and/or positive CT scan results. Controls were included based on negative PCR results and without positive CT scan results. All the patients hospitalised during the initial phase were excluded from the outpatient population analysis. Thus, we started this study with 410 outpatients (150 cases and 260 controls).

The patients were contacted by phone after November 2021: the time range between the infection and the follow-up was 14 to 20 months. Initial questions were established on the symptoms presented on the day of call and vaccination status. Quality of life was assessed using the EQ-5D-5L scale with a telephone version validated by the EuroQol group [17]. Data collection by phone was more relevant for this multicentre research during the COVID time because of several lockdowns and travel restrictions.

Secondary exclusion criteria were unreachable patients and those with communication problems. Among the control population, we also asked and excluded patients who presented a positive test for COVID-19 (PCR, antigen, and/or serology) between the initial consultation and telephone call.

### 2.2. Main Outcomes

We considered the health-related quality of life assessed using the EQ-5D-5L scale as the main outcome. The EQ-5D-5L is a generic questionnaire that is not specific to a particular pathology. This scale is simple and quick to administer and includes five dimensions: mobility, autonomy, daily activities, pain or discomfort, and anxiety or depression. Each dimension was evaluated on a scale of 1 to 5 (1: no problems, 2: slight problems, 3: moderate problems, 4: severe problems, and 5: extreme problems/unable to). This scale is supplemented by a subjective quality of life score (EQ-EVA) from 0 (“the worst possible health”) to 100 (“the best possible health”). 

The EQ-5D-5L is one of the most commonly used instruments for measuring the health-related quality of life in clinical research. This instrument was translated into French and confirmed by the EuroQol group [17].

### 2.3. Statistical Analysis

Using the EQ-5D-5L scale, we evaluated the health status of the patients from 1 to 5 in five domains and from 0 to 100 with the EQ-EVA. This health state classifier can describe health states that are often reported as vectors ranging from 11111 (full health) to 55555 (worst health). Numerous societal value sets have been derived from population-based valuation studies of a country, when applied to the health state vector, result in a preference-based score that typically ranges from states worse than dead (<0) to 1 (full health), anchoring dead at 0. For example, “11112” meaning slight problems in the anxiety or depression dimension and no problems in any of the other dimensions, is associated with a EQ-5D-5L score of 0.929 in France.

The number of subjects required was calculated assuming a mean score of 0.86 with a variance of 0.2^2^ and tolerance of 0.04 to the mean. It was estimated to be 49 individuals per group, or 98 individuals in total. This hypothesis was based on a French study comparing the post-COVID quality of life in two groups [15].

We estimated the proportion of people lost to follow-up to be between 25% and 55%, according to studies evaluating quality of life by phone call [15,18].

Continuous variables are presented as means and standard deviations, and categorical variables are presented as numbers and proportions.

To compare categorical variables between the two groups, a Chi^2^ test was used, or a Fisher’s exact test was used when the former was not applicable (theoretical numbers less than five). For continuous variables, we performed Student’s *t*-test in the case of a normal distribution or Wilcoxon rank-sum test.

All statistical tests were two-sided, and statistical significance was set at *p* < 0.05. Data were analysed using Statistical Analysis System.

### 2.4. Regulatory Procedures

This was a non-interventional study involving a human patient (RIPH). Procedures regulated by the “Jarde law” are required to guarantee the protection of people participating in research. The agreement of the French Data Protection Authority (National Commission on Informatics and Liberty) was obtained for reference methodology MR-003. This methodology governs research including health data with a character of public interest, and patients consented to participation after being informed. This study was approved by the French Institutional Review Board.

## 3. Results

### 3.1. Sample Characteristics

**Figure 1:**
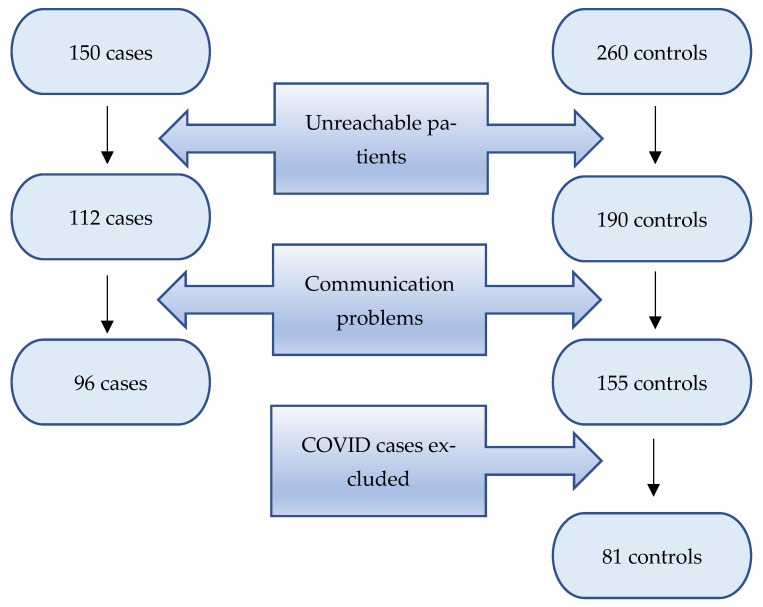
Flowchart.

The study population included 177 patients (96 cases and 81 controls; Figure 1).

We were able to reach 73% of the patients via phone call and obtained 43% complete responses to the questionnaire. Epidemiological and clinical characteristics of the study population are shown in Table 1.

The two groups were comparable in terms of age and sex (no statistically significant difference, *p* > 0.05). There was no significant difference in COVID vaccination between the two groups. Hwever, there was a significant difference in cardiological history (*p* = 0.005) and obesity (*p* = 0.013).

COVID-19 outpatients had significantly higher fatigue (39.6%), dyspnoea (24%), chest pain (8.3%), and ageusia (5.21%) one year after SARS-CoV-2 infection.

### 3.2. Quality of Life

Regarding our main outcome, the EQ-5D-5L index mean (SD), which evaluates quality of life in five domains, was at 0.87 (0.19) in cases and 0.95 (0.11) in controls (*p* = 0.002). The EQ-VAS mean (SD), which subjectively evaluates quality of life, was at 78 (17.6) in cases and 86.7 (9.7) in controls (*p* < 0.001).

Thus, outpatient cases had a significantly lower health-related quality of life than controls one year after SARS-CoV-2 infection.

### 3.3. The 5 Domains of EQ-5D-5L Score

Of the COVID-19 cases, 47.9% reported problems in at least one of the EQ-5D-5L dimensions compared with 35.8% of the controls, with 6.3% and 2.5% reporting severe or extreme problems, respectively.

**Figure 2:**
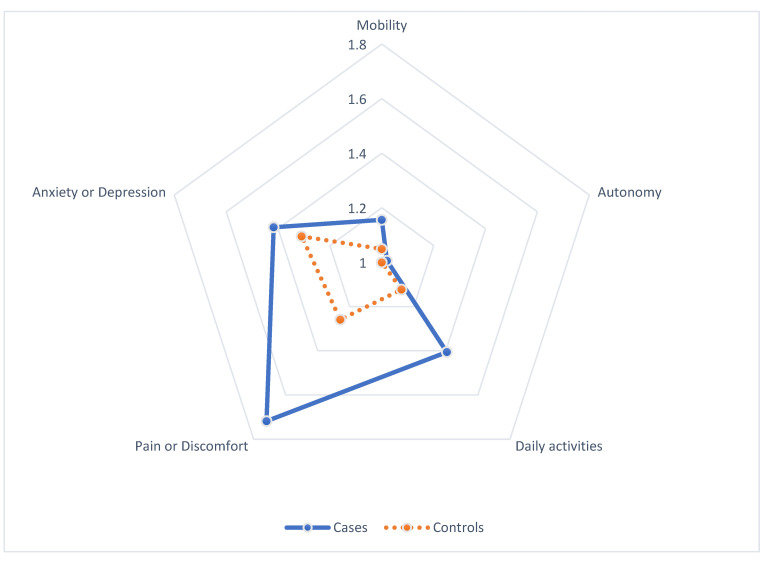
Each domain of EQ-5D-5L score.

The distribution of the EQ-5D-5L scores in the five domains showed a significant difference in pain or discomfort (*p* < 0.001) and daily activities (*p* = 0.01) (Figure 2).

Approximately 43% of the cases complained of a problem in the area of pain or discomfort compared to 21% of the controls. Regarding daily activities, approximately 23% of the cases complained of a problem compared to 6% of the controls.

However, there were no significant differences in autonomy, mobility, and anxiety or depression between the two groups. More than 20% of the patients in the two groups were complained of anxiety or depression.

## 4. Discussion

### 4.1. Main Outcome 

The main objective of this study was to assess the long-term health-related quality of life in outpatients with COVID-19. One year after SARS-CoV-2 infection, outpatients cases had significantly lower health-related quality of life than controls with an EQ-5D-5L index mean of 0.87 and an EQ-EVA mean of 78; alteration was more intense in pain or discomfort and daily activities.

### 4.2. Comparison with Literature

These COVID-19 ambulatory patients may have an impaired quality of life after SARS-CoV-2 infection compared like initially hospitalised patients. In 2020, in France, the EQ-5D-5L index mean was 0.86, and the EQ-EVA mean was 70.3 among 120 patients more than 100 days after hospitalization [15]. However, this study included a few severe patients because those hospitalised directly in the ICU were excluded. Thus, the alteration in the quality of life post-COVID does not only concern hospitalised patients, but also ambulatory patients who should not be neglected.

Lower values are found in other countries, such as the United Kingdom [19], Norway [20], and Iran [14] with EQ-5D-5L index of 0.72, 0.69, and 0.61, respectively. However, the post-COVID evaluation period was much shorter (between 1 and 3 months), which could have influenced the quality of life. In the United Kingdom and Norway, these lower values can also be explained by the characteristics of patients who initially present with severity criteria (ICU admission). The lower EQ-5D-5L index value in Iran (middle-income country) compared to the UK and Norway (high-income country) may be due to better health services of rich countries rather than patient characteristics, such as age (average 58.4 years in Iran vs. 70.5 years in the UK) or disease severity (18% of patients in Iran vs. 32% in the UK in the ICU).

In ambulatory care, we also found lower values in Belgium [21]: the EQ-5D-5L index mean was 0.63 and the EQ-VAS mean was 51.3 less than 3 months after confirmed SARS-CoV-2 infection. These low values can be explained by the method of selection made from a Facebook group that included 1200 patients with persistent symptoms following an episode of COVID-19, which underestimates the quality of life in an ambulatory population. We also noted that the most affected areas were pain or discomfort, and daily activities, as we found in our study. This is more valuable as this “long COVID” population, which complains of persistent symptoms, is overrepresented here. Management of these patients should focus on the assessment of pain or discomfort, and daily activities.

### 4.3. Persistent Symptoms

The most frequently reported symptoms were fatigue (39.6%) and dyspnoea (24%), which was also found in other studies [15,19], and the presence of these symptoms one year after SARS-CoV-2 infection in outpatients was notable.

The prevalence of fatigue is consistent with previous outbreaks of SARS, H1N1, and Ebola, in which a large proportion of patients with fatigue were found eligible for a diagnosis of chronic fatigue syndrome/myalgic encephalomyelitis [22]. The prevalence of dyspnoea is comparable to that reported in a meta-analysis, in which 11% to 45% of SARS and MERS survivors suffered from dyspnoea for up to 12 months [23].

Fatigue and dyspnoea are likely to contribute to the reduced ability to perform daily activities observed in this cohort of COVID-19 outpatients.

### 4.4. Anxiety or Depression

Our results showed that more than one-fifth of the patients complained of anxiety or depression in both cases and controls, with no significant difference. Anxiety or depression probably do not explain the deterioration in quality of life observed in these cases. The presence of this problem in cases and controls can be explained by the impact of the COVID-19 pandemic on the mental health of the general population [24,25].

Therefore, caution is needed before categorising all patient’s post-COVID complaints as “functional”.

### 4.5. Determinants

Regarding the characteristics of the two groups, a significant difference was found in the cardiological history and obesity. All others characteristics didn’t show a significant difference between the two groups. The statistical comparison with a control group rule out the influence of external factors on quality of life. 

Biological tests were not available during the initial phase of the epidemic. According to the recommendations in force, they are mainly used in high-risk cases. This may explain the predominance of certain comorbidities among cases compared to controls.

This factors alone (cardiological history and obesity) probably do not explain the difference of quality of life between the two groups. Indeed, the previous SARS and MERS outbreaks, which used the SF-36 to measure health-related quality of life, showed significantly poor quality of life at 1 year, which is lower than the quality of life of those affected by chronic diseases (using normative data) [26]. The previous publications in 2020 and 2021 also showed a lower post-COVID quality of life in different populations of several countries. Thus, the impaired quality of life observed in outpatient cases likely reflects the impact of COVID-19.

## 5. Strengths and Limitations

There are many retrospective studies on the consequences of COVID-19, but few prospective cohorts and even fewer comparative prospective cohorts with long-term follow-ups. To our knowledge, this study is the first to show an alteration in the quality of life one year after infection with SARS-CoV-2 in an ambulatory population through a statistical comparison with an uninfected control group. This statistical comparison with a control group is fundamental to rule out the influence of external factors on quality of life, as our way of life has been upset by this pandemic (confinement, wearing a mask, social distancing, etc.).

The strength of our study lies in its multicentre and prospective nature. Cases were selected during the first wave based on a positive COVID test (PCR or CT scan) and not on suspicion of COVID, which limits the risk of selection bias. We used a reliable scale (EQ-5D-5L score) to assess quality of life because it is a commonly used instrument, validated in clinical research, and translated into French with reference values, allowing us to compare our results with other studies. This questionnaire is standardised and administered by a single contact, which limits measurement bias.

Our study had several limitations. We obtained a little less than half (43%) of the answers to the questionnaire, although corresponding to our initial estimate, which represents a high rate of inaccessible patients. Phone calls as a method of contact limited the ability to contact some participants such as people with dementia, speech difficulties, and non-French speakers. Our sample was not representative of the original population. In addition, the case population had more comorbidities (cardiological history and obesity) than the controls because of an initial sorting of the use of a PCR test according to the severity criteria. Thus, our results must be confirmed in larger cohorts to be able to be extrapolated to the general population.

This outpatient study only included patients over 18 years of age, while persistent symptoms were also described in the paediatric population [27].

Patients were infected during the first wave of the epidemic in 2020, but the virus is constantly gaining mutations, which can lead to changes in its internal characteristics, such as virulence or contagiousness [28], as well as in its clinical expression [29]: therefore, we can question the reproducibility of the results with the new variants.

## 6. Conclusions

This study is the first to show an alteration in the quality of life of COVID-19 outpatients after one year. This study suggests an impact of COVID-19 on the long-term quality of life of outpatients and hope to improve the management of long COVID. Appropriate guidance and community rehabilitation programs are required for outpatients with persistent symptoms of COVID-19. Research must continue to confirm these results in larger cohorts.

## Figures and Tables

**Table 1:** Symptoms and quality of life of 177 patients at 1 year after their visit in a COVID ambulatory center.

		Cases	Controls	*p* Value
		n = 96	n = 81	
Age (years)		45.8 (14.9)	43.2 (14.5)	0.242
Sex (male)		39 (40.6)	30 (37.0)	0.625
Vaccination		87 (90.6)	77 (95.0)	0.260
Comorbidities				
	Cardiological	22 (23.1)	5 (6.94)	0.005
	Respiratory	9 (9.5)	11 (15.3)	0.253
	Diabetes	4 (4.2)	4 (5.6)	0.727
	Obesity	11 (11.8)	1 (1.4)	0.013
	Tobacco	7 (7.9)	8 (11.2)	0.463
	Immune deficiency	8 (8.4)	5 (6.9)	0.724
Symptoms				
	Fatigue	38 (39.6)	17 (21)	0.008
	Cough	11 (11.5)	8 (9.9)	0.734
	Dyspnea	23 (24)	3 (3.7)	<0.001
	Chest pain	8 (8.3)	1 (1.2)	0.032
	Anosmia	9 (9.4)	2 (2.5)	0.058
	Ageusia	5 (5.21)	0 (0)	0.037
	Throat sore	4 (4.2)	2 (2.5)	0.534
	Headaches	12 (12.5)	4 (4.9)	0.081
Quality of life				
	EQ-5D-5L index	0.87 (0.19)	0.95 (0.11)	0.002
	EQ-EVA	78 (17.6)	86.7 (9.7)	<0.001
	*Results are expressed as count (%) for categorical variables and as mean (standard derivation) for continuous variables.*			

## Data Availability

The data presented in this study are available on reasonable request from the corresponding author.
